# Assessing community awareness and participation in health facility governing committees in two districts of Tanzania: a cross-sectional study

**DOI:** 10.1186/s13690-024-01415-0

**Published:** 2024-10-29

**Authors:** Hussein Athuman Kapuya, Stephen Oswald Maluka, Anna-Karin Hurtig, Miguel San Sebastian

**Affiliations:** 1 Dar es Salaam University College of Education, University of Dar es Salaam, Dar es Salaam, Tanzania; 2 Department of Epidemiology and Global Health, Umeå University, Umeå, Sweden

**Keywords:** Community participation, Social accountability, Health facility governing committees, Health system, Tanzania

## Abstract

**Background:**

Tanzania has been promoting community participation in health, either directly or through health facility governing committees (HFGCs), as part of its wider, ongoing health system reforms since the 1990s. Although some studies have assessed the functioning of the HFGCs, little is known about community knowledge and involvement in their activities.

**Methods:**

A cross-sectional survey assessing community awareness and participation in HFGCs was conducted between July and October 2022, involving two rural districts of Tanzania, which were selected based on their performance: Handeni, showing low performance and Mbarali, high performance. A total of 1,184 household heads living in the catchment areas of diverse facilities were involved. Frequencies and percentages were calculated overall and by district. The Chi-squared test was applied to assess statistically significant differences in knowledge, awareness, and participation between the districts.

**Results:**

The results revealed that 85.3% of the participants were unaware of the presence of the HFGCs and 91.7% did not know their roles. Additionally, 87% had neither heard nor seen any announcement about the selection of committee members. Only 14.5% considered that HFGCs were accountable to the community. While 96.7% of the respondents said they had never received any feedback from the HFGCs, only 8.1% reported that HFGCs were collecting views from the community. Regarding participation, 79.9% believed that the community had not been supporting their activities; however, 44.7% believed that the committees were important in improving health service delivery. Feedback and support were more common in Handeni.

**Conclusion:**

The level of community awareness of and participation in HFGCs was very low in both districts. The Ministry of Health and the President’s Office of Regional Administration and Local Government should implement an action plan to raise community awareness of the role of the HFGCs and their significance in promoting social accountability within the Tanzanian health system.

**Table:** 

**Text box 1. Contributions to the literature**
• How well the HFGCs can promote social accountability in the local health systems depends on community awareness and support. Not many studies have assessed how much the communities in rural Tanzania know about HFGCs and participate in their activities.
• In general, the vast majority of community members are not aware of HFGCs and do not participate in committee activities, which limits their ability to contribute to improving the health system in Tanzania.
• There is an urgent need for the government to step in and implement a plan to increase public awareness of HFGCs and encourage greater community involvement in the committees.

## Background

Community participation in health is fundamentally about community involvement in health governance [1], and it is considered a prerequisite for attaining universal primary health care [1–3]. Involving the community through health committees in the delivery of primary health care (PHC) helps bridge the gap between communities and health facilities, making services more responsive to community needs [4].This participation creates an enabling environment for those typically excluded due to factors such as gender, ethnicity, or class [5]. To enable the community to work effectively with the committee, it is important to build a strong relationship by ensuring they understand the committee´s role, who its members are, and informing them how to utilize and contact the committee [6]. Additional steps include mobilizing the community, seeking their input and assistance, involving them in some activities, and having the committee provide feedback to them [6]. The significant global movement towards promoting community participation in health either individually or through health committees can be traced back to the Alma Ata Declaration of 1978. In its fourth article, the Declaration states that “people have the right and duty to participate individually and collectively in the planning and implementing of their health care plans” [7]. In many low- and middle-income countries, particularly in Africa, the adoption of health facility committees was one of the mechanisms used to promote community engagement in primary healthcare facilities [8]. These initiatives emerged strongly with the Bamako initiative of 1987, which called upon African member states to establish at least one component of community participation such as village health committees, community health workers (CHWs) and health centre committees [9]. Numerous experiences have reported how these committees have positively contributed to improving health outcomes and have additionally increased the transparency, community participation and responsiveness of the health system [4, 10].

Like other sub-Saharan African countries, Tanzania has been promoting community participation through various forms of health committees as part of its wider ongoing health system reforms since the 1990s. To ensure participation in the implementation of primary healthcare (PHC), the National Health Policy of 1990 made it mandatory for each village to have at least two CHWs who would serve as a link between the community and the health system. The CHWs at the village level were introduced to give the communities more power and voice in the ownership of health facilities [11]. To further involve the community in the management of PHC services, the government established guidelines for the establishment and functioning of the Council Health Service Board (CHSB) and the health facility governing committees (HFGCs) in the early 2000s [12]. The CHSB and the HFGCs were formally given the mandate to oversee the management and provision of PHC services. The 2001 guideline was revised in 2013 to accommodate reforms in the health sector over the past two decades, such as requiring the community to participate in the activities of the HFGCs by identifying challenges, seeking for funds, hold health service providers accountable and participate in the planning and budgeting for their facilities. Members of the HFGCs should be elected by the community through the general assembly, and the elected members must work with the community and build a supportive relationship [13] HFGC members, particularly those elected by the community, should also be accountable to their respective community and ensure that they consult the community before their meetings and provide feedback after their meetings to enable the community to be aware of what is going on in their PHC facilities [13].

Previous studies in Tanzania on the functioning of the HFGCs have noted that the committees were performing poorly in carrying out their duties [10, 14–16]. Even community participation in managing the facilities was limited to a few local actors, such as the council health management teams and medical professionals, while leaving other stakeholders, including the community, powerless [7, 17]. Low funding, a lack of fiscal autonomy, late transfer of funds to the facility, and a lack of community participation in planning have also been reported as major impediments to the implementation of decentralization at PHC facilities [16, 18, 19]. To address such challenges, especially restricted budgetary autonomy and power, the government introduced the Direct Health Facility Financing (DHFF) reform in 2017/2018 [8, 20]. Under the DHFF, funds from the government, as well as from other sources such as users’ fees, insurance schemes, and development partners are directly deposited to the bank account of each facility (dispensary, health centre, and district hospital) [8]. Under this arrangement, the HFGCs and service providers, thus have considerable autonomy to plan, budget and manage facility finances to improve the delivery of healthcare services [8, 20].

While there has been some research assessing the functioning of the HFGCs, including after the introduction of the DHFF [8, 10, 20–22], no studies have assessed community knowledge and involvement in the activities of the HFGCs. The functioning of the health facility committee is highly dependent on the extent to which community members are aware, knowledgeable, and actively participate in their activities. This study, therefore sought to assess community knowledge and awareness of the HFGCs and their participation in these committees´ activities, in rural Tanzania.

## Methods

### Study context

The study involved two districts, Handeni and Mbarali, located in the North-Eastern and South-Western parts of Tanzania, respectively.The two districts were purposively selected based on their performance in the 2018 Star Rating assessment conducted by the Ministry of Health [23]. Handeni was rated as a low-performing district, while Mbarali was rated as a high-performing one. Handeni, one of the 11 districts of the Tanga region, covers 355,702 km^2^ and has a total population of 276,646. The major economic activities in the district include livestock farming, hunting, and gathering, fishing, transportation, business, forestry resources, and hunting and gathering. The district has one hospital, one health centre and five dispensaries [24]. Mbarali is one of the seven districts of the Mbeya Region. In 2016, there were 331,206 people in the district [25]. The main economic activities in Mbarali include agriculture (it is famous for rice farming), fishing, business, transportation, forestry resources, hunting and gathering [26]. The district has one hospital, five public health centres, and 34 dispensaries.

### Study design

This cross-sectional survey was carried out between July and October 2022. The study was part of a bigger project related to social accountability of the health system at the local level in Tanzania, implemented by the Dar es Salaam University College of Education (DUCE) in collaboration with Umeå University, Sweden, and the President’s Office Regional Administration and Local Government (PO-RALG) in Tanzania.

### Sampling and sample size

This study used a multistage non-probability sampling technique in selecting the facilities and respondents. First, a similar number of facilities were selected in both districts by selecting one hospital from each district, two health centres in Mbarali, and the only health centre in Handeni. To balance the number of facilities at the dispensary and health centre levels, four dispensaries were selected in Mbarali and five dispensaries were selected from Handeni. Then, a list of geographically accessible villages in the catchment area of these facilities was prepared, giving a total of 31 villages (*mitaa*), 15 (48.4%) from Handeni and 16 (51.6%) from Mbarali. A total sample size of 1,184 households was estimated for both districts based on a power of 80%, a significance level of 5%, the prevalence of any of the outcomes of 50% and a potential non-response rate of 10%. The sample size was estimated according to Cochran’s formula using a two-step procedure [27]; first the sample size was calculated using the unlimited formula, followed by the finite formula (see below).

$$n\, = \,{{{z^2}\, \times \,\widehat p\left( {1\, - \,\widehat p} \right)} \over {{{\rm{\varepsilon }}^{\rm{2}}}}}\, \to \,n'\, = \,{n \over {1\, + \,{{{z^2}\, \times \,\widehat p\left( {1\, - \,\widehat p} \right)} \over {{{\rm{\varepsilon }}^{\rm{2}}}N}}}}$$

where z = 1.96; p̂ = 50%, ε = 5% and N = total district population.

This gave a total sample size of 1184 households for both districts. A potential non-response rate of 10% was also considered in the calculations. A sampling interval (n^th^) for each village was then calculated by dividing the population in each respective district by the total population of each village/mtaa under study. The number of households to be involved in each village/mtaa was obtained by dividing the total population of each village/mtaa by the sampling interval. The starting point of each village was identified with the help of the village leaders, and households were selected until the desired sample size in each village under study was reached. All selected households participated in the study.

### Data collection

A team of trained research assistants was used to administer the questionnaire to the selected heads of households (man or woman); whichever household head was available and interested in participating was given a questionnaire. Different questions were used to capture the awareness of the community about the presence of the HFGCs and participation in their activities. The survey gathered information on eight themes (dependent variables): (i) community awareness of the HFGCs; (ii) community knowledge of the roles of the HFGCs; (iii) community support for the activities of the committees; (iv) accountability of the members of the committees to the community; (v) community participation in selecting the members of the committees; (vi) feedback provided on the activities of the committees; (vii) collection of views from the community; and (viii) the perceived importance of the committees. Participants were required to answer “Yes”, “No” or “I don’t know”. The questionnaire also included questions about the socio-demographic information of the participants such as gender, age, level of education, and occupation.

The questionnaire gathered additional information on the socio- economic and demographic characteristics of respondents (independent variables). Sex, was categorized as men and women, and age was divided into four groups:21–30, 31–40, 41–50, and 51–60 years. Education level was classified as none, primary, secondary and tertiary levels. Occupation was categorized as farmer/pastoralist, business, retired and other economic activities which include fishing, transportation, hunting and gathering and forestry resource. Additionally, the type of health facility was categorized into dispensaries, health centers, and district hospitals. The districts were also classified by their performance, with Handeni identified as low-performing and Mbarali as high-performing.

### Data analysis

Information was collected in paper form and entered into an Excel spreadsheet, and moved to Stata for analytical purposes. Data were analysed descriptively by calculating frequencies and percentages in total for each one of the eight dependent variables and according to district (the independent variable). Chi-squared tests were applied to assess statistically significant differences in the outcomes between the districts using a 95% confidence interval for inferential purposes.

### Ethical clearance

Ethical approval to conduct the study was granted by the National Institute for Medical Research (NIMR) in Tanzania, with certificate No: NIMR/HQ/R.8a.Vol. IX/3928. Permission to conduct the study was also obtained from the President’s Office Regional Administration and Local Government (PO-RALG), the Regional Administrative Secretary, and District Executive Directors in the respective districts. Participants were approached to seek their informed consent to participate in the study. Verbal consent was preferred, because, based on our experience, asking respondents in rural areas to sign formal consent forms would intimidate them into participating in the study. All participants approached agreed to participate willingly.

## Results

Table one presents the socio-demographic characteristics of the participants in total and by district.The majority were women (68.5%), aged above 50 years (31.5%). Regarding socio-economic level, most of the participants had finished primary education (66.0%) and were employed in farming or pastoralism (71.5%). Other economic activities include fishing, transportation, hunting and gathering, and forestry resource 58(7.5%). Meanwhile,247 (20.9%) were engaged in business and 31(2.6%) were retired. Handeni accounted for 43.5% and Mbarali for 56.5% of the participants (See Table 1).

**Table 1:** Socio-demographic characteristics of the respondents in total and by district (*N* = 1184), 2022

	Total *N* (%)	Handeni *n* (%)	Mbarali *n* (%)
**Sex**			
Men	373(31.5)	196(38.1)	177(26.5)
Women	811(68.5)	319(61.9)	492(73.5)
**Age of respondents**			
20–30	230(19.4)	100(19.4)	130(19,4)
31–40	291(24.6)	117(22.7)	174(26.1)
41–50	290(24.5)	122(23.7)	168(25.1)
>50	373(31.5)	176(34.2)	197(29.4)
**Level of education**			
Never been to school	185(15.6)	99(19.2)	86(12.9)
Primary	781(66.0)	319(61.9)	462(69.1)
Secondary	178(15.0)	73(14.2)	105(15.7)
Tertiary	40(3.4)	24(4.7)	16(2.4)
**Occupation**			
Farming or pastoralism	847(71.5)	342(66.4)	505(75.5)
Business	247 (20.9)	107(20.8)	110(16.4)
Retired	31(2.6)	21(4.1)	10(1.5)
Other activities	58(7.5)	45(8.7)	44(6.6)
**District**			
Total	1184 (100)	515 (43.5)	669 (56.5)

### Community awareness and participation in HFGC activities

As shown in Table 2 and 85.3% of the participants were unaware of the presence of HFGCs. In addition, 91.7% did not know the roles of the HFGCs and 88.3% had neither heard about any announcement for selection nor participated in selecting the committee members.

**Table 2:** Prevalence of community awareness of the HFGCs and their participation in their activities in total and by district (*N* = 1184), 2022

	Total *N* (%)	Handeni *n* (%)	Mbarali *n* (%)	*P*-value
Are you aware of the presence of the HFGC?				
Yes	174 (14.7)	81 (15.7)	93 (13.9)	
No	1010 (85.3)	434 (84.3)	576 (86.1)	0.30
Do you know any roles of the health facility governing committee/Board?				
Yes	98 (8.3)	46 (9.0)	52 (7.8)	
No	1086 (91.7)	469 (91.0)	617 (92.2)	0.35
Do you think the community supports the activities undertaken by the HFGCs? *				
Yes	238 (20.1)	125 (24.3)	113 (16.9)	
No	946 (79.9)	390 (75.7)	556 (83.1)	0.001
Do you think the HFGC are accountable to the community?				
Yes	172 (14.5)	74 (14.4)	98 (14.6)	
No	456 (38.5)	218 (42.3)	238 (35.6)	
I don´t know	556 (47.0)	223 (43.3)	333 (49.8)	0.95
Have you ever heard/seen any announcement on the selection of HFGC members?				
Yes	139 (11.7)	64 (12.4)	75 (11.2)	
No	1045 (88.3)	451 (87.6)	594 (88.8)	0.41
Have you ever received any feedback on the activities of the HFGCs? *				
Yes	39 (3.3)	23 (4.5)	16 (2.4)	
No	1145 (96.7)	492 (95.5)	653 (97.6)	0.03
Do HFGC members collect views from the community before their meetings?				
Yes	96 (8.1)	45 (8.7)	51 (7.6)	
No	590 (49.8)	278 (54.0)	312 (46.7)	
I don´t know	498 (42.1)	192 (37.3)	306 (45.7)	0.54
Do you think the HFGCs are of any importance?				
Yes	529 (44.7)	230 (44.7)	299 (44.7)	
No	131 (11.1)	48 (9.3)	83 (12.4)	
I don´t know	524 (44.2)	237 (46.0)	287 (42.9)	0.94

* Statistically significant differences between districts

Regarding the accountability of the HFGCs to the community, only 14.5% of the participants thought that the committees were accountable to the community. In addition, 96.7% of the respondents said they had never received any feedback from the HFGCs, and nearly half (49.8%) of the respondents reported that HFGCs were not collecting views from the community. While 44.7% of the respondents thought that HFGCs were important, 79.9% had the view that the community was not supporting the activities of the HFGCs.

No statistical differences were found across six of the eight outcomes between the two districts. Despite the low prevalence of positive answers, respondents from Handeni (low performing district) reported a statistically significantly better response in the items of feedback received from the HFGCs compared to Mbarali.

## Discussion

This study assessed community knowledge and awareness of the HFGCs and their participation in the activities of the committees in the Handeni and Mbarali districts of Tanzania. The findings revealed that most of the participants in both districts were unaware of the presence of the health committees and their roles. Also, participants felt that the community was not participating in the committee´s activities and that committees were not accountable to the community, neither through collecting views nor providing feedback. Despite nearly half of the participants acknowledging the committees´ importance, they were not actively supporting them .Furthermore, participants indicated that they were not involved in electing committee members.

The lack of knowledge among participants about the HFGCs and their roles was surprising, given the efforts of the government in promoting community participation in health over the past two decades through the committees to promote social accountability [13]. The government requires the committees to ensure the community is well engaged in identifying problems, setting priorities, monitoring, and evaluating the services provided at their facilities [13]. Lack of community awareness and participation in the committees indicates that the committees lacked capacity and good strategies to make themselves known and engage the community in their activities [6]. It is also an indication of deficient support from the district councils, which were required by the guidelines to ensure that the HFGCs were created to empower the community in managing the delivery of their health services [13].The councils were required to support the committees through training to make them understand their roles through the CHMTs [13].The literature indicates that the first step in building a working relationship between the community and the committees is to make sure the community knows what a health committee is, what it can do, and how the community can make use of the committee [6] .Several examples of how to make the community aware of the committee have been reported in some previous studies. In a study from Kenya, nearly half (44.5%) of the facility users involved in the study were aware of the presence of the health committees [28]. The study reported that some facilities, especially in rural areas, had the names of the facility committee members displayed so they were visible to the users of the facility. Another study from the same country similarly showed that community awareness about the health committees and their roles could be raised by disseminating information through the CHWs or the use of public noticeboards at facilities [29].A study from Cape Town Metropole, South Africa, suggested that strengthening the relationship between the committees and the community involves raising awareness about the committees, their functions, and involving the community in their activities [30].It further, emphasized the need to build the capacity of the committee members to perform their roles effectively by providing training on community participation, good governance, monitoring and evaluation to promote participatory roles [30]. Another study from Zambia also recommended that continuous training and mentoring are key to building capacity and enhancing the sustainability of the committees [31].

It was also unexpected that the community had not been providing support to the committees in their activities. The guidelines for the establishment and functioning of the CHSBs and HFGCs require that the community, under the leadership of the Mtaa/Village Executive Officer, support the committees in managing the facilities by participating in a number of their activities, such as implementing the annual collaborative health centre plans or the participatory annual service delivery plans [32, 33]. In addition to their limited knowledge about their roles, the HFGCs´ low impact on the community and their inability to mobilize the community in their activities could further explain this finding.It explains why despite many community members acknowledging the importance of the committees, they were not participating in their activities in any way. A systematic review from sub-Saharan African countries noted that the low impact of the committees on life in the community made community members not support the committees, as they viewed them as bodies designed to service the health centres, not the community [34]. Similarly, a study from Ghana showed that committed health committees were able to secure support from the community and stakeholders both locally and internationally [35].These members led fundraising efforts to finance activities like renovating buildings and purchasing medical equipment like x-rays and other equipment. They organized resource mobilization events and door-to - door campaigns, sought medical assistance from pharmaceutical companies, and contacted individuals abroad for support for their facilities [35].

Closely interlinked with the lack of knowledge and awareness, the findings of this study revealed that HFGC members in both districts were not accountable to the community in any of the examined dimensions. They were neither collecting views from the community before their meetings nor providing feedback after their meetings, contrary to the guidelines regulating their establishment and operation [13]. Listening to the communities is important, as it ensures that the committees work on real community concerns, and providing feedback is a mechanism for improving both community understanding and the responsiveness of the health system [28]. The failure of the HFGCs to listen to and inform the communities indicate that the committees neglected to take any initiative to be accountable to the community as stated in the guidelines [13]. The findings of this study are consistent with a previous study conducted in Mukuranga, Tanzania, which observed that one way to improve the functioning of the committees was to ensure they were adequately and regularly sensitized about being active and responsive to the community needs [36].Similarly, findings from Nepal indicated that the committees´ failure to collect views from the communities they represent implied that they were not reflecting community concerns [37]. In contrast, a study from Kenya shows that the health workers were able to address the relevant community concerns because the committee members brought information from the community, which could not be possible without their involvement [38].

Furthermore, lack of community participation in selecting their representatives to the committees helps to partly explain why the community was not aware of the committees and their functions. Evidence shows that this limitation makes health committees to become invisible to the community, resulting in a poor understanding of the committees and their roles [1]. Similarly, this lack of participation explains why committee members were not accountable to the community. An appropriate selection process is a key initial step in determining the committees functioning and members´ legitimacy [37]. Committees that function properly normally have general elections where community members elect their representatives [30]. These results are consistent with other studies conducted within and outside Africa. A study from South Africa noted that the formation of health committees through the appointment of members or through election by a few community members resulted in a weak link between the community and the committees [1]. Another study from Zambia also recommended that elections for members of the committees can be an effective mechanism to increase accountability for health institutions at the community level [31]. While a study from Nepal indicated that appointed members by politicians or facility managers were often not accountable to the wider community [37].

## Methodological consideration

The questionnaires were designed according to the best practices recommended in the national health policy of 2007 and the 2013 guidelines that govern the functioning and operation of the committees as well as community roles in the committees.The study, conducted in two rural districts in Tanzania, may provide insights applicable to other districts and regions in the country and similar developing countries facing comparable socio-economic and healthcare challenges. While the large sample size and lack of attrition contribute to the internal validity of the study, the selection of villages close to the facilities might have introduced a certain bias, possibly overestimating some responses.Even so, given that only two districts were included in the study, the generalizability of the results to other districts in the region or other regions of the country or other low-and middle-income countries with similar characteristics should be undertaken cautiously.While careful training was provided to the data collectors, interview and response bias could possibly have occurred, given the self-reported nature of the study design. The extent of the influence of these potential biases on the findings is, however, difficult to assess.

## Conclusion

The level of community awareness and participation in the activities of the HFGCs in the study districts was very low, which limits the achievement of the goal of social accountability in the Tanzanian health system. While having a legal framework is necessary, this study has shown that it is not sufficient and proposes three key actions. First, health committee members should actively cultivate strong working relationships with the community as per guidelines governing their functioning and operations [13]. Secondly, to ensure that health committee members are known as legitimate representatives of the community, the procedures indicated in the guidelines for selecting the committee members should be observed [13].Third, the district councils through their CHMTs should train the HFGC members to understand the importance of cooperating with and being accountable to the community they represent as per guidelines governing their functioning and operations [13].Additionally, the community should be educated by the local government leaders on the importance of collaborating with the committees and holding them accountable.In all of these educational processes, consideration should be given to culturally adapted and appropriate communication and social marketing tools.

## Data Availability

No datasets were generated or analysed during the current study.

## Abbreviations

CHSBs: Council Health Service Boards
CHWs: Community Health Workers
DHFF: Direct Health Facility Financing
DUCE: Dar es Salaam University College of Education
HFGCs: Health Facility Governing Committees
NIMR: National Institute for Medical Research (NIMR)
PHC: Primary Health Care
PO-RALG: President´s Office Regional Administration and Local Government
